# Gastric Outlet Obstruction Secondary to Severe Thoracolumbar Scoliosis

**DOI:** 10.7759/cureus.51753

**Published:** 2024-01-06

**Authors:** Nikitha Boyapati, Anand Trivedi

**Affiliations:** 1 Acute Surgical Unit, Fiona Stanley Hospital, Perth, AUS

**Keywords:** degenerative spine disease, idiopathic, thoracolumbar, gastric outlet obstruction, scoliosis

## Abstract

A 78-year-old woman with a history of idiopathic thoracolumbar scoliosis presented with signs, symptoms, and imaging findings consistent with a gastric outlet obstruction secondary to the rib cage impinging on the pylorus of the stomach. She underwent an operative intervention and intra-operative findings were consistent with severe scoliosis with the right rib cage impinging on the pylorus, causing gastric outlet obstruction. A laparoscopic procedure was performed to pexy the greater curvature of the stomach to the left upper quadrant and a percutaneous endoscopic trans-gastric jejunostomy was inserted at the end.

Thoracolumbar idiopathic scoliosis is a relatively benign common condition. However, with the increasing aging population and resultant higher incidence of progression to degenerative scoliosis, more patients are presenting with severe spinal and rib cage deformities that can cause rare intra-abdominal sequelae. We report the first case of a gastric outlet obstruction caused by the rib cage impinging on the pylorus in a patient with severe thoracolumbar scoliosis.

## Introduction

Gastric outlet obstruction (GOO) is a mechanical obstruction of the pylorus, distal stomach, or duodenum [1]. This results in the prevention or reduction of gastric emptying and causes nausea, vomiting, and abdominal pain [1]. The exact incidence of GOO is not known, but it is estimated to be 5% of all bowel obstructions [2]. It is caused by malignant and benign conditions.

The most common cause of GOO is gastric or periampullary (cholangiocarcinoma, duodenal adenocarcinoma, and pancreatic adenocarcinoma) malignancies [3]. Other malignant causes include lymphoma, hepatocellular carcinoma, lymphoma, and metastases to proximal small bowel [4]. Benign causes of GOO include peptic ulcer disease, caustic ingestion, superior mesenteric artery syndrome, adhesions, and strictures [3]. We report the first case of GOO caused by the rib cage impinging on the pylorus in a patient with severe thoracolumbar scoliosis.

## Case presentation

An independent 78-year-old woman presented to the emergency department with a one-month history of generalized colicky abdominal pain. She reported associated nausea, loss of appetite, and constipation. Her past medical history includes severe thoracolumbar scoliosis, large abdominal wall hernia, and iron deficiency anemia. She previously had an open hysterectomy and open appendicectomy. On examination, she was alert and mildly uncomfortable due to the pain. She was afebrile, normotensive, and saturating well on room air with a regular pulse of 80. Abdominal examination demonstrated a soft but large abdominal hernia with complete loss of the anterior abdominal wall. She was distended but minimally tender around the umbilicus.

**Figure 1:**
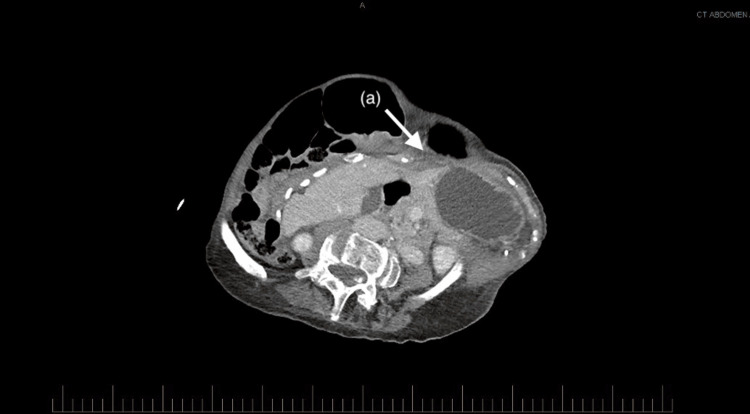
Axial slice of a CT abdomen and pelvis (a) Gastric outlet obstruction due to impingement by the right chest wall

**Figure 2:**
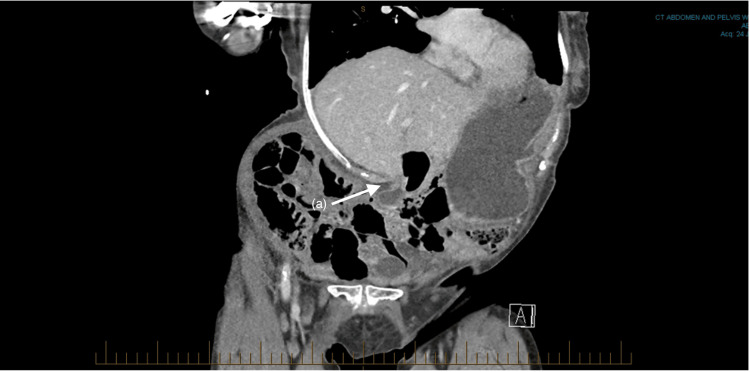
Coronal slice of a CT Abdomen and Pelvis. (a) Gastric outlet obstruction due to impingement by the right chest wall

Her hematological investigations were unremarkable, and she had a lactate of 0.9 mmol/L. A computed tomography (CT) of her abdomen and pelvis with intravenous contrast was performed to investigate the cause of her pain. It demonstrated a gastric outlet obstruction due to effacement of the pylorus between the distended gastric antrum and the right lower chest wall (Figures 1, 2).

Following the investigations, she was diagnosed with a gastric outlet obstruction secondary to severe thoracolumbar scoliosis and was admitted to the general surgical unit for further management. Initial management included the insertion of a nasogastric tube and fluid resuscitation, and she was kept nil by mouth. On admission, she was proactively reviewed by a consultant geriatrician. The geriatrician ensured her nutrition was optimized by organizing a pre-operative iron transfusion and pre-emptively involved allied health to assess and begin optimizing her mobility status. She underwent operative management of the gastric outlet obstruction under general anesthesia within 24 hours of admission. The operative findings included severe scoliosis with the right rib cage impinging on the pylorus causing gastric outlet obstruction and the pedunculated border of the left liver lobe was folded over on itself over the top of the pylorus. The procedure was performed laparoscopically with ports in the right upper and left upper quadrants. The pedunculated liver lobe was untwisted with immediate release of the pylorus and then four intra-corporeal sutures were used to pexy the greater curvature of the stomach to the left upper quadrant abdominal wall. Finally, a gastroscopy was done, and a percutaneous endoscopic trans-gastric jejunostomy was inserted.

Postoperatively, she had a slow recovery and required a period of inpatient rehab. However, there were no postoperative complications, and she was discharged 21 days after the operation.

## Discussion

Thoracolumbar scoliosis is defined as a spinal deformity that results in lateral curvature and rotation of the thoracic and lumber vertebrae within the curve [5]. The causes of scoliosis include neuromuscular, syndrome-related, idiopathic, and congenital amongst others [5]. The most common cause is idiopathic and is defined as a curvature of the spine in the frontal plane or a Cobb angle that is greater than 10 degrees [5,6]. The majority of idiopathic scoliosis cases arise during adolescence and have an incidence of up to 2% [7]. It is significantly more common in women and women are 10 times more likely to have moderate idiopathic scoliosis where the Cobb angle is greater than 30 degrees [7].

Idiopathic scoliosis tends to progress in adult life and risk factors for curve progression have been extensively researched. It has been found that age is a significant risk factor along with bone mineral density, initial curve magnitude, and skeletal maturity [6]. A systematic review by Lenz et al. found that patients with a Cobb angle of greater than 25 degrees were more likely to sustain curve progression with a Cobb angle of greater than 50 degrees [6]. Skeletal maturity is another important risk factor, idiopathic scoliosis is more likely to progress in individuals that have not achieved skeletal maturity [8]. With age and no treatment, patients with idiopathic scoliosis progress to develop adult degenerative scoliosis due to asymmetrical disc and facet joint degeneration [9]. This ultimately results in the accentuation of the spinal curvature, which increases the patient’s Cobb angle.

Patients with idiopathic scoliosis often present with dissatisfaction with personal appearance, back pain that progresses with age, and pulmonary symptoms like shortness of breath [10]. Treatment for scoliosis is based on the type of scoliosis, severity of curve magnitude, and skeletal maturity [5]. Treatment options include observation, bracing, and surgery. Observation and bracing are reserved for skeletally immature patients, whereas surgery is offered in skeletally mature patients [5]. In older adults who have progressed to degenerative scoliosis, surgery involving long-segment fusion has been shown to have the best outcome [9]. Non-operative measures like multi-modal analgesia, which ranges from anti-inflammatory analgesia to nerve blocks are considered in degenerative scoliosis patients if they are unfit for spinal corrective surgery [9].

In this case, the patient had idiopathic scoliosis since she was an adolescent. She did not undergo any bracing or surgical correction for it, as she was growing up and hence it likely progressed with skeletal maturity and age to a level that resulted in a severe deformity. This severe curvature of the thoracolumbar spine results in an asymmetrical rib cage which contributes to causing a gastric outlet obstruction. The surgery performed on her was minimally invasive and was aimed are relieving the obstruction. She was considered too frail for extensive spinal and or intra-abdominal surgery to correct her deformity.

## Conclusions

This case emphasizes the need for general surgeons to be vigilant for the abdominal complications of idiopathic thoracolumbar scoliosis. Idiopathic thoracolumbar scoliosis is a relatively common condition and with the aging population, there will be a larger proportion of patients who will progress to adult degenerative scoliosis. Severe spinal curvature, especially of the thoracic spine, can cause rib asymmetry to affect the size and shape of the intra-abdominal cavity. That can ultimately affect intra-abdominal organs and result in conditions like gastric outlet obstruction due to mechanical impingement.
